# Chebulinic acid derived from triphala is a promising antitumour agent in human colorectal carcinoma cell lines

**DOI:** 10.1186/s12906-018-2412-5

**Published:** 2018-12-27

**Authors:** Min Wang, Yanru Li, Xianda Hu

**Affiliations:** 1 Department of Rheumatology, Peking Union Medical College Hospital, Chinese Academy of Medical Sciences and Peking Union Medical College, Beijing, China; 2 College of Chemistry and Molecular Engineering, Peking University, Beijing, China; 3 Beijing Tibetan Hospital, China Tibetology Research Center, 218 Anwaixiaoguanbeili Street, Beijing, 100029 Chaoyang China

**Keywords:** Triphala, Colon cancer, Chebulinic acid, Chebulagic acid

## Abstract

**Background:**

Triphala is an Ayurvedic *rasayana* formulation reputed for its antitumour activities, and chebulinic acid and chebulagic acid, along with other phenolic acids, have been proposed to be responsible for its effects.

**Methods:**

In this study, the anti-proliferative activities of these agents were evaluated in colorectal carcinoma cell lines with three phenotypes exposed to several batches of triphala samples with different quantities of chebulinic acid and chebulagic acid. The pro-apoptotic and anti-migratory activities and the probable antitumour mechanisms of the more potent anti-proliferative phytochemical were also investigated.

**Results:**

The results demonstrated that chebulinic acid, which exerts potent anti-proliferative, pro-apoptotic and anti-migratory effects, is a key molecule for maintaining the antitumour efficacy of triphala. The antitumour mechanism of chebulinic acid is probably related to the PI3K/AKT and MAPK/ERK pathways.

**Conclusions:**

Chebulinic acid is not only a critical component of the anticancer activities of triphala but also a promising natural multi-target antitumour agent with therapeutic potential.

## Background

Colorectal cancer refers to malignant tumours that begin in cells of the large intestine, colon and rectum. Despite advances in diagnosis and treatment in past decades, colorectal cancer remains a significant health concern and substantial socioeconomic burden [1]. It has been reported that colorectal cancer is the third most common and fourth most deadly human malignancy globally [2].

Triphala is an Ayurvedic herbal formula consisting of three myrobalan fruits, namely, *Phyllanthus emblica* Linn.*, Terminalia chebula* Retz., and *Terminalia bellirica* (Gaertn.) Roxb., in equal proportions [3]. Triphala was first recorded in the Ayurvedic text *Charaka Samhita* and has been used for various diseases for thousands of years [4]. Triphala has been used extensively not only in Ayurvedic medicine but also in many other traditional medical practices that are influenced by Ayurveda, such as Tibetan, Thai and Unani medicines.

Recent clinical studies have proved that triphala has a wide spectrum of biological activities, including laxative, antimicrobial, immunomodulatory, and antioxidative activities, and is effective for constipation, gingivitis, arthritis, cataracts, and several other diseases or disorders [5–9]. Accumulating experimental evidence has also suggested that triphala is a promising herbal formulation for cancer therapy. It has been reported that triphala not only shows chemo-, radio- and oxidant-protective activities, which indicates that triphala has the potential to prevent oncogenesis [10–12], but also exhibits marked anti-proliferative and apoptosis-inducing properties against different tumour cell lines and animal models without causing damage to normal cells [13, 14]. Moreover, triphala can regulate angiogenesis and epithelial-to-mesenchymal transition to suppress tumour invasion and metastasis [15, 16].

Pharmaceutical analyses have revealed that triphala is rich in saponins, terpenoids, tannins, flavonoids and phenolic acids [17]. Among these compounds, ellagitannins and tannin-related compounds, especially gallic acid, ellagic acid, chebulinic acid and chebulagic acid, are considered the major constituents of the bioactivities of triphala [18–20]. However, the poor drug absorption and low bioavailability of gallic acid and ellagic acid has led to an increasing number of studies focused on chebulic ellagitannins [21, 22]. Both chebulinic acid and chebulagic acid have been demonstrated to contribute to the antitumour activities of triphala, but chebulinic acid shows higher antitumour activities than gallic acid, ethyl gallate, luteolin, and tannic acid against a human osteosarcoma cell line in vitro [23]. It has also been reported that chebulinic acid has the highest antioxidative activity among all the constituents of triphala [24]. Nevertheless, it remains difficult to determine whether chebulic ellagitannins are an important antitumour component, since interactions among the complex chemical components of triphala could result in synergistic or inhibitory effects that may influence antineoplastic activity. Previously, we obtained a batch of inferior triphala with a desired signature characterized by lower contents of chebulinic acid and chebulagic acid, which provides us with an opportunity to evaluate whether chebulinic acid is the major antitumour constituent of triphala.

## Methods

### Drugs and chemicals

The superior triphala preparation, which contains large amounts of chebulinic acid and chebulagic acid, was manufactured by Dabur India Ltd. (Alwar, India) with a batch number of AL1675, while the inferior triphala preparation containing lower levels of chebulinic acid and chebulagic acid was provided by Beijing Tibetan Hospital, China Tibetology Research Center. Standard analytical grade chebulinic acid and chebulagic acid were procured from Shanghai Yuanye Biological Technology (Shanghai, China).

### Preparation of triphala extract

The superior and inferior triphala extracts were prepared following the same procedure. Finely powdered triphala was extracted by ultrapure water with refluxing for 1 h. The extract solution was centrifuged at 4000 rpm for 15 min at room temperature. Then, the supernatant was filtered through a 0.45 μm membrane filter (Merck Millipore Ltd., Cork, Ireland) to remove particulate matter. The solvent was removed by rotary evaporation followed by freeze-drying. The achieved extract powder was weighed and stored at − 20 °C. The yields of the superior and inferior triphala extracts were 6.85 and 7.25%, respectively.

### High-performance liquid chromatography (HPLC) analysis

The HPLC analysis was performed on a Waters Alliance 2695 system (Waters Corp, Milford, USA) equipped with a Waters 2489 UV detector. The dried triphala extract was dissolved in water, injected into a Waters XBridge Shield RP_18_ column (250 mm × 4.6 mm × 5 μm) and eluted with a linear gradient from 15 to 80% aqueous acetonitrile solution. The absorbance was detected at 254 nm.

### Cell culture

The human colorectal carcinoma cell lines HR8348, LoVo, and LS174T were purchased from the China Infrastructure of Cell Line Resources and cultured in RPMI 1640 medium (Corning Inc., Corning, USA) supplemented with 10% foetal bovine serum (FBS) (Corning) and 1% penicillin-streptomycin (Beyotime Biotechnology Inc., Nantong, China) in a standard humidified incubator (NuAire Inc., Plymouth, USA) at 37 °C under a 5% CO_2_ atmosphere.

### Cell proliferation assay

To evaluate cytotoxicity, cell viability was assessed by cell counting kit (CCK)-8 assays following the manufacturer’s protocol (Dojindo Inc., Kumamoto, Japan). Briefly, the colorectal carcinoma cells were seeded in 96-well plates at a density of 1 × 10^4^ cells per well for 24 h and cultured with increasing concentrations of drugs (25–150 mg/L) or chemicals (20–70 μmol/L) for an additional 48 h. The tetrazolium salt-based CCK-8 solution was then added to each well, and the cells were incubated for another 3 h. The optical density (OD) was measured at 450 nm.

### Cell apoptosis assay

To examine apoptosis, DNA fragmentation immunoassays were performed using the Cell Death Detection ELISA Plus kit (Roche Diagnostics, Basel, Switzerland) according to the manufacturer’s specifications. Briefly, the cells were seeded and cultured with the same approach described in section 2.5. After incubation, the cells were collected by trypsinization, resuspended in phosphate-buffered saline (PBS), lysed with lysis buffer and centrifuged successively. The supernatants containing cytoplasmic histone-associated DNA fragments were used for the quantitative sandwich enzyme immunoassay. The OD was determined at 405 nm.

### Transwell migration assay

To detect metastatic ability, Boyden chamber assays were performed using the Transwell system (Corning) according to the manufacturer’s instructions. Briefly, 5 × 10^4^ colorectal cancer cells in serum-free RPMI 1640 medium were added to the upper layer of the cell-permeable membrane of a 24-well Transwell plate, while the lower chamber was filled with complete medium with FBS. Following 6 h of incubation, the cells that migrated through the micropores of the membrane to the lower chamber of the Transwell plate were stained using 0.1% crystal violet. The OD was determined at 630 nm.

### Western blotting assay

The colorectal carcinoma cells were cultured in 10 cm petri dishes and grown to approximately half confluence and then treated with chebulinic acid at the IC_50_ concentration for 48 h. The cultured cells were collected and lysed in radioimmunoprecipitation assay (RIPA) buffer supplemented with protease inhibitor cocktail and phosphatase inhibitor cocktail (Sigma-Aldrich Corp., St Louis, USA). The concentration of extracted protein was measured using a Pierce BCA Protein Assay Kit (Thermo Fisher Scientific, Waltham, MA). After sodium dodecyl sulfate-polyacrylamide gel electrophoresis (SDS-PAGE) and transfer to a nitrocellulose membrane, the protein was reacted with monoclonal antibodies against AKT, ERK1/2 (Cell Signaling Technology, Danvers, USA) and caspase-3 (Abcam, Cambridge, UK) and subsequently labelled with secondary antibodies (Abcam), which were developed through enhanced chemiluminescence (ECL) using the Pierce ECL Plus Western Blotting Substrate (Thermo Fisher Scientific).

### Statistical analysis

All data represent the mean ± standard deviation (SD) of at least three independent experiments. Statistical analyses were performed by one-way ANOVA with the least significant difference (LSD) post hoc test for multiple comparisons using SPSS Statistics 19.0 software (IBM, Chicago, USA), and *p* values less than 0.05 were considered statistically significant.

## Results

### The superior triphala preparation exhibits more potent anti-proliferative activities than the inferior triphala preparation against colorectal carcinoma cells

The cell viability effects of the superior and inferior triphala preparations on the human colorectal cancer cell lines HR8348, LoVo, and LS174T were investigated by a CCK-8 assay. The half maximal inhibition concentration (IC_50_) values were calculated by probit analyses. The results showed that the superior triphala preparation had outstanding growth inhibition activities against all three tumour cells, which were significantly and positively correlated with the concentration (Fig. 2a). The IC_50_ values in HR8348, LoVo, and LS174T cells were 85.57 ± 5.73, 90.28 ± 8.20 and 84.50 ± 3.56 mg/L, respectively. In contrast, the inferior triphala preparation showed distinctly weaker cytotoxic effects on tumour cells (Fig. 2b), with IC_50_ values of 127.24 ± 6.09, 129.28 ± 8.12 and 131.46 ± 6.42 mg/L, (Table 1) respectively, in the same cell lines.

**Table 1:** Half maximal inhibition concentration (IC50) values of triphalas and chebulic ellagitannins

cell lines	A	B	C	D	E	F
HR8348	85.57 ± 5.73	127.24 ± 6.09	81.19 ± 6.05	119.31 ± 4.57	37.18 ± 2.89	51.74 ± 2.32
LoVo	90.28 ± 8.20	129.28 ± 8.12	87.34 ± 4.62	116.27 ± 5.48	40.78 ± 2.61	56.31 ± 4.77
LS174T	84.50 ± 3.56	131.46 ± 6.42	80.66 ± 6.38	118.82 ± 4.20	38.68 ± 2.12	53.53 ± 0.65

The IC50 (mg/L) of superior triphala (A), inferior triphala (B), inferior triphala with chebulinic acid (C), inferior triphala with chebulagic acid (D), chebulinic acid (E) and chebulagic acid (F)

### The most important differentiating factor between the superior and inferior triphala preparations is the content of chebulinic acid and chebulagic acid

**Fig. 1:**
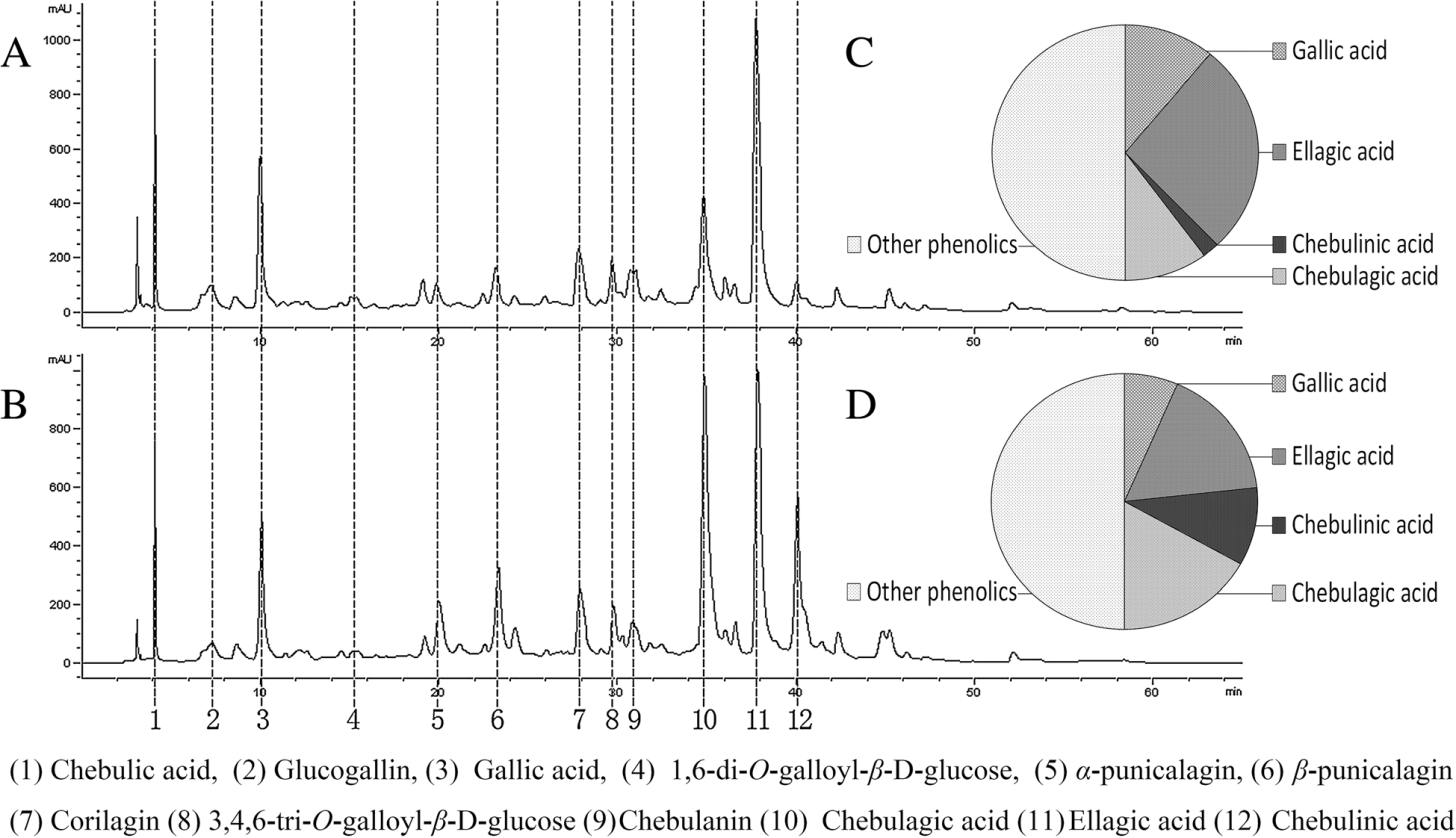
Comparison of the contents of components between the superior and inferior triphala extracts, as determined by HPLC. **a** HPLC chromatogram of the superior triphala preparation; **b** HPLC chromatogram of the inferior triphala preparation; **c** approximation of the contents of the superior triphala preparation based on the area percent by HPLC; and **d** approximation of the contents of the inferior triphala preparation based on the area percent by HPLC

Quantitative analyses of the triphala compositions were performed by HPLC. The peaks were identified by aligning the retention time and peak shape with published data [25]. The amount of each component was estimated and compared using the area normalization method, and chebulinic acid and chebulagic acid were further quantified using the external standard method. The HPLC results illustrated that the compositions of the two triphala preparations were identical, whereas their relative contents were different (Fig. 1). The contents of chebulinic acid and chebulagic acid in the superior triphala preparation were 1.91 and 5.52 times higher than those in the inferior triphala preparation, respectively, while the levels of ellagic acid and gallic acid were 1.40 and 1.47 times lower than those in the inferior triphala preparation, respectively. There is sufficient evidence to prove that ellagic acid and gallic acid are antineoplastic. Predictably, the lower anti-proliferative activities of the inferior triphala preparation may result from the reduction in chebulinic acid and/or chebulagic acid content.

### Chebulinic acid is more effective than chebulagic acid against the proliferation of colorectal carcinoma cells

**Fig. 2:**
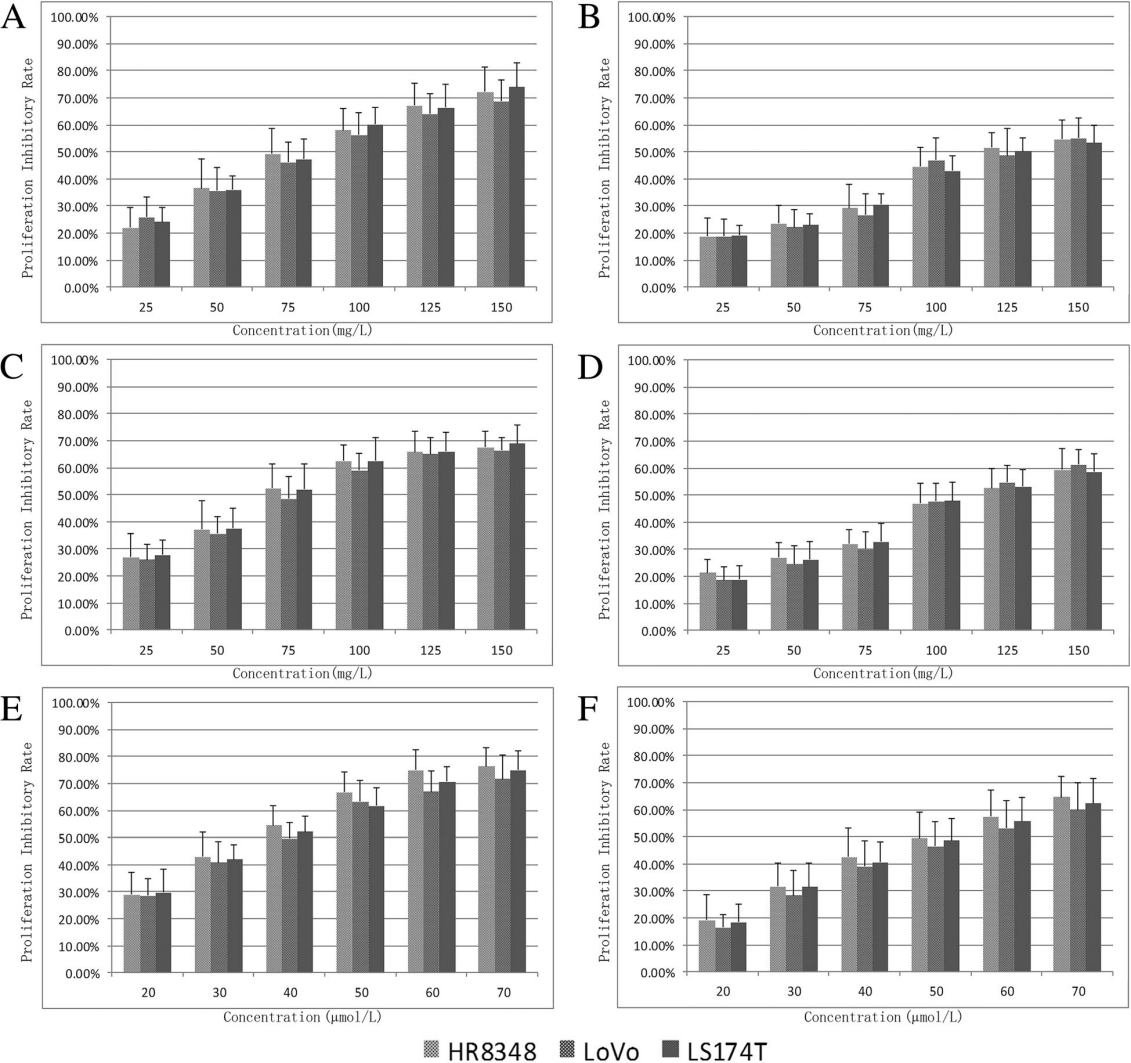
Proliferation inhibition rates of different human colorectal carcinoma cells treated with **a** superior triphala (higher chebulinic acid, higher chebulagic acid); **b** inferior triphala (lower chebulinic acid, lower chebulagic acid); **c** inferior triphala with corrected chebulinic acid (higher chebulinic acid, lower chebulagic acid); and **d** inferior triphala with corrected chebulagic acid (lower chebulinic acid, higher chebulagic acid) or **e** chebulinic acid or **f** chebulagic acid at concentrations of 25, 50, 75, 100, 125, and 150 mg/L for 48 h. The relative IC_50_ values were estimated with regression analysis by probit using SPSS and presented in Table 1

The anti-proliferative activities of chebulinic acid and chebulagic acid were also investigated. The CCK-8 experiment results showed that although both chebulinic acid (Fig. 2e) and chebulagic acid (Fig. 2f) were able to restrain cell proliferation at low concentrations, the bioactivity of chebulinic acid is obviously higher than that of chebulagic acid. The IC_50_ values of chebulinic acid against HR8348, LoVo, and LS174T cells were 37.18 ± 2.89, 40.78 ± 2.61 and 38.68 ± 2.12 μmol/L, respectively, while the same values were 51.74 ± 2.32, 56.31 ± 4.77 and 53.53 ± 0.65 μmol/L, respectively, for chebulagic acid (Table 1).

### Supplementation of the inferior triphala preparation with chebulinic acid, but not chebulagic acid, is able to significantly improve its anti-proliferative activities

To further determine the importance of chebulinic acid and/or chebulagic acid for the antitumour activities of triphala, chebulinic acid and chebulagic acid were added separately to the inferior triphala preparation in the correct proportion, according to the contents in the superior triphala preparation. The cytotoxic activities were detected by the aforementioned method. The results showed that the addition of chebulinic acid to the inferior triphala preparation reinforces its anti-proliferative activities against the colorectal carcinoma cell lines HR8348, LoVo, and LS174T (Fig. 2c), with IC_50_ values of 81.19 ± 6.05, 87.34 ± 4.62 and 80.66 ± 6.38 mg/L, respectively. However, the addition of chebulagic acid alone was unable to significantly elevate the observed antitumour activities (Fig. 2d). The IC_50_ values of the inferior triphala preparation supplemented with chebulagic acid were 119.31 ± 4.57, 116.27 ± 5.48 and 118.82 ± 4.20 mg/L, respectively (Table 1). These results indicated that chebulinic acid is a vital component of triphala for restraining the proliferation of the above human colorectal carcinoma cell lines.

### Chebulinic acid is a promising pro-apoptotic and anti-metastatic agent

**Fig. 3:**
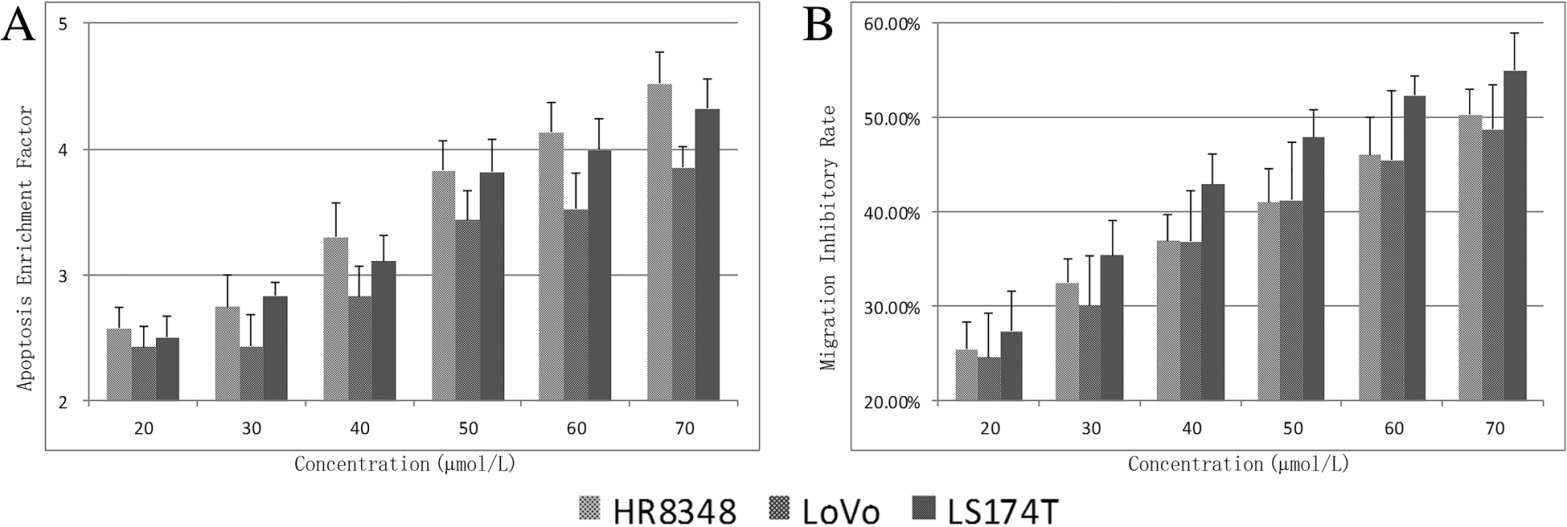
Effects of chebulinic acid on cell apoptosis and migration in HR8348, LoVo, and LS174T cells. **a** Determination of apoptosis by a DNA fragmentation immunoassay. The enrichment factor was used as an index of apoptosis, which represents the level of DNA fragmentation in cells treated with chebulinic acid relative to that in untreated cells. **b** Examination of cell migration capacity through a Boyden chamber assay. The number of migrated cells was counted by absorbance after crystal violet staining. The inhibitory rates were calculated as percentages with respect to the control

The induction of apoptosis in the human colorectal cancer cell lines HR8348, LoVo, and LS174T after treatment with chebulinic acid was evaluated by enzyme-linked immunosorbent assay. The oligo-nucleosome enrichment of mono- and oligo-nucleosomes released into the cytoplasm was calculated as the ratio of the absorbance of treated cells to the absorbance of the corresponding negative control. The results demonstrated that chebulinic acid was able to induce programmed cell death significantly (Fig. 3a).

The inhibitory effects on motility were investigated by a Transwell migration assay. The effects of chebulinic acid on the migration of colorectal carcinoma cells were expressed as the ratio of the absorbance of treated cells to that of the control cells. The results revealed that chebulinic acid treatment induced a marked reduction in cellular migration towards the lower chamber (Fig. 3b).

### The antitumour effects of chebulinic acid are related to the activation of caspase-3 and the downregulation of ERK pathways

**Fig. 4:**
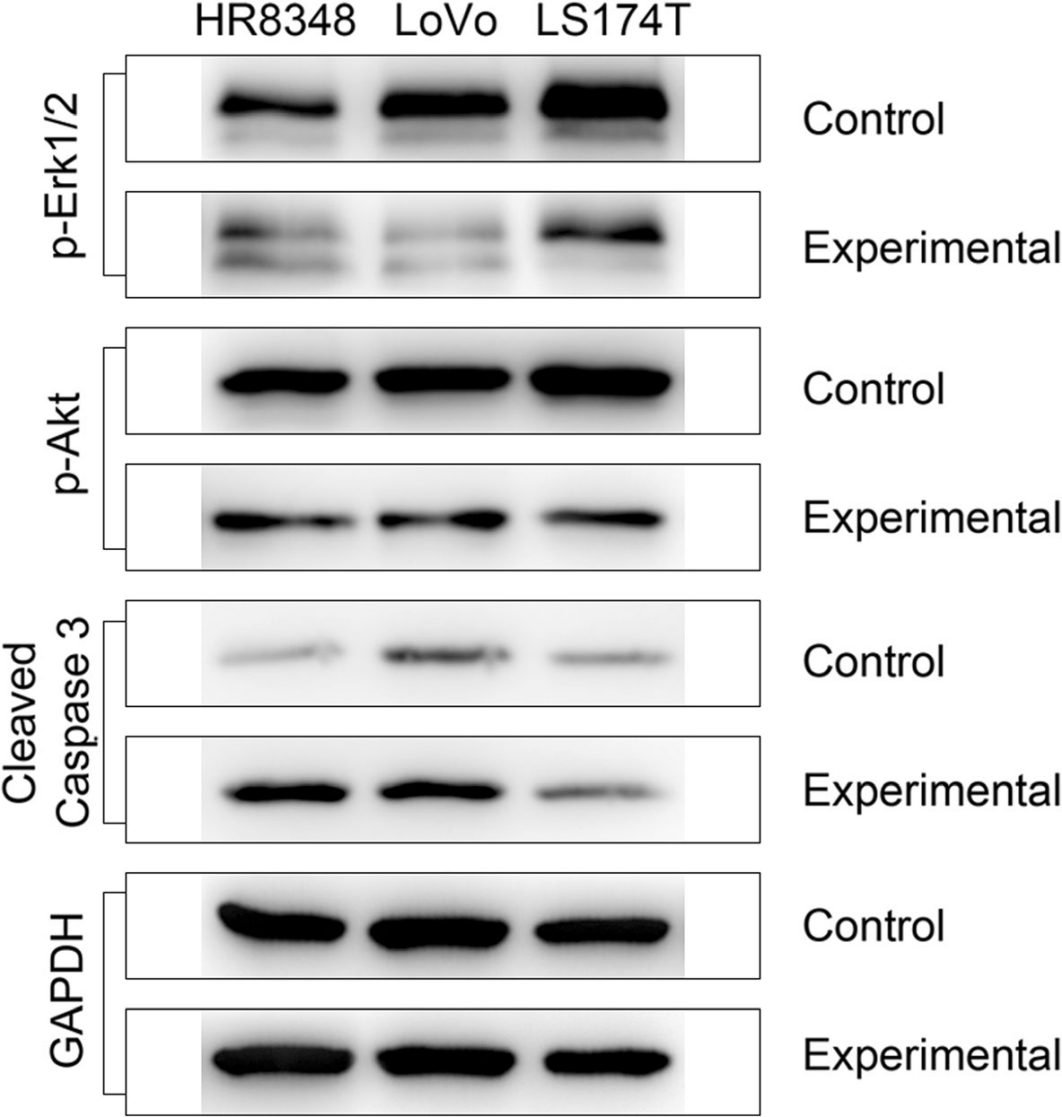
Analyses of apoptosis-related proteins in HR8348, LoVo, and LS174T cells by Western blotting. The cells were treated with the IC_50_ of chebulinic acid for 48 h. Phosphorylated ERK and AKT and cleaved caspase-3 were detected using the corresponding antibodies. GAPDH was used as a loading control

To investigate the molecular mechanism of chebulinic acid, Western blotting experiments were carried out to measure the levels of related proteins. The results showed that the degrees of p-ERK1/2 and p-AKT were decreased by 1.39- and 1.32-fold in HR8348 cells, 1.96- and 1.33-fold in LoVo cells, and 1.28- and 1.34-fold in LS174T cells, respectively, while the content of the cleaved caspase-3 protein was increased by 3.26-, 1.40-, and 1.37-fold in HR8348, LoVo and LS174T cells, respectively (Fig. 4). This finding suggests that the antineoplastic mechanism of chebulinic acid may be related to the activation of caspase-3 and the downregulation of the AKT and ERK pathways.

## Discussion

Colorectal cancer is one of the most frequent and impactful cancers worldwide. Colorectal cancer also accounts for a significant and growing proportion of all cancer cases diagnosed in hospitals. Triphala, which is renowned for its colon cleansing and detoxification effects, has also been reported as a potent natural formulation that is effective against colorectal carcinoma stem cells and therefore has clinical value for the prevention and treatment of colon cancer [11]. In this study, the dose-dependent tumour-growth inhibitory activities of triphala on different human colorectal carcinoma cell lines were confirmed by CCK-8 cytotoxic assays. However, a notable difference in antitumour effects was also observed between the two kinds of triphala, which was subsequently confirmed by HPLC analyses and attributed to variations in their component contents.

Among all discovered constituents of triphala, chebulinic acid, which exerts the most significant antitumour and antioxidant activities in vitro, has long been recognized as one of the most abundant and effective monomers [21, 22]. However, due to the synergistic and antagonistic effects between the compounds present in the triphala, it is difficult to determine whether chebulinic acid is necessary for triphala bioactivity. Herein, we tested the anti-proliferative activities of a triphala preparation with higher chebulinic acid and chebulagic acid contents, a triphala preparation with lower chebulinic acid and chebulagic acid contents, and triphala preparations with corrected chebulinic acid or chebulagic acid contents in human colorectal carcinoma cells. The results demonstrated that the absence of chebulinic acid and chebulagic acid significantly affected the anti-growth effects of triphala, and these decreased anti-proliferative activities could be corrected by supplementation with chebulinic acid but not chebulagic acid. Moreover, chebulinic acid alone has been proven to be more effective than chebulagic acid. By comparing the above results, we concluded that chebulinic acid is a critical component that may play a dominant role in the anti-proliferation activity of triphala against colorectal cancer cells in vitro. Subsequent studies also revealed that chebulinic acid is a natural apoptosis-inductive and metastasis-inhibitive product.

**Fig. 5:**
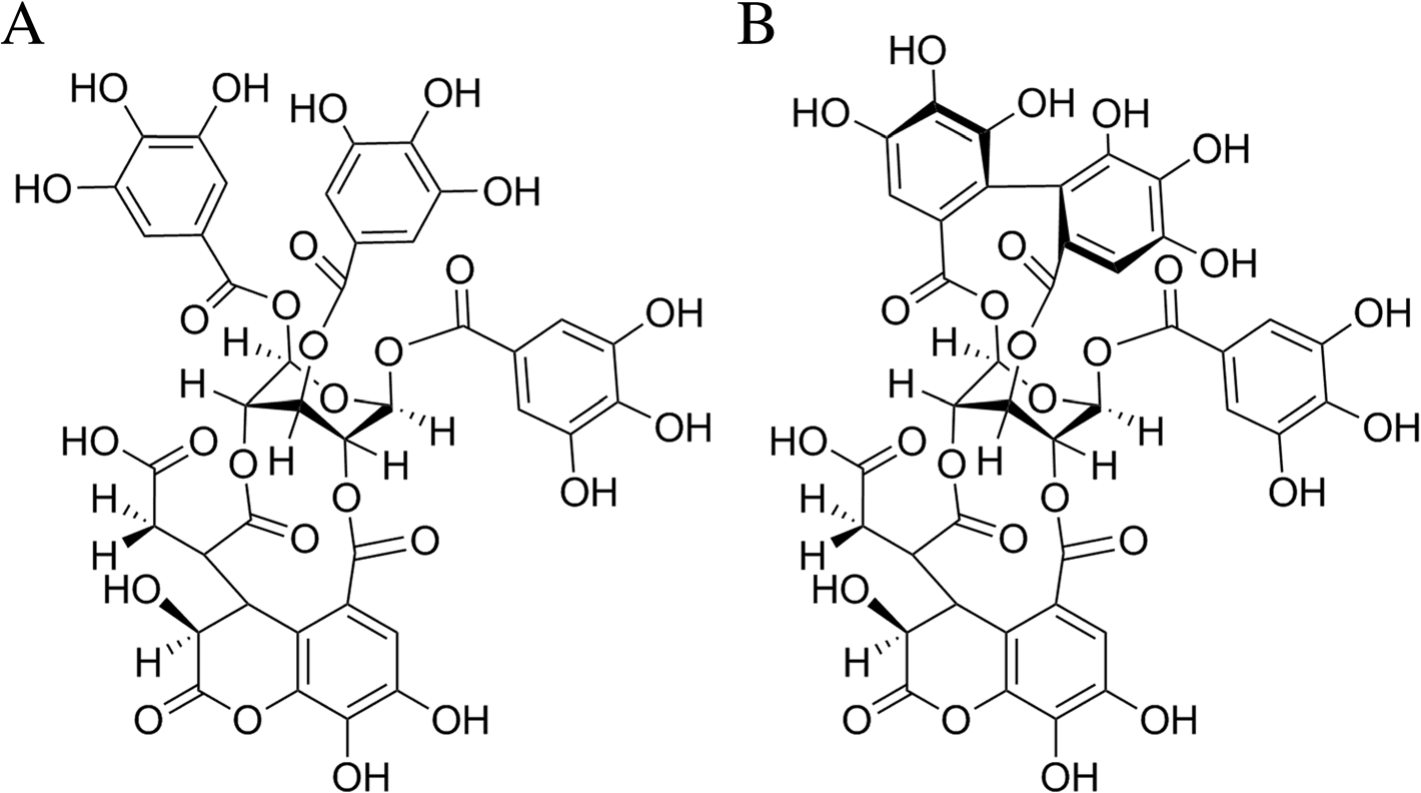
Structures of chebulinic acid and chebulagic acid according to a previous study [26]. **a** chebulinic acid (1,3,6-tri-O-galloyl-2,4-O-chebuloyl-β-D-glucopyranoside); **b** chebulagic acid (1-O-galloyl-2,4-O-chebuloyl-3,6-O-HHDP-β-D-glucose)

Chebulinic acid and chebulagic acid share the same molecular backbone of a *β*-D-glucose residue linked to a chebuloyl moiety (Fig. 5). The difference between the molecules is that chebulinic acid has three free galloyl groups attached to the *β*-D-glucose residue, while chebulagic acid contains only one galloyl group and a hexahydrodiphenoyl (HHDP) group, which is assumed to be a substitution for the two galloyl groups [25]. The presence of the constrained HHDP group in chebulagic acid is considered to result in larger spatial hindrance and less molecular flexibility [24, 26]. Chebulinic acid, in contrast, which has a more favourable structure for entering the binding cavities or catalytic pockets of target proteases or enzymes, usually shows more broad-spectrum and potent biological activities (with the exception of chelation activity) [27, 28].

To date, chebulinic acid has been studied for its antioxidative, anti-pathogenic, anti-inflammatory, anti-fibrotic and antitumour activities [29–31]. Although the detailed mechanisms remain unclear, there is no doubt that chebulinic acid produces efficacy through multiple targets simultaneously. A series of tumour-associated proteins, such as vascular endothelial growth factor A (VEGF-A), small mothers against decapentaplegic homologue 3 (SMAD-3), matrix metalloprotease 2 (MMP-2), GATA-binding protein 2 (GATA2), and regulator of G protein signalling 7 and 8 (RGS-7/8), have been found or assumed to be related to the antitumour activities of chebulinic acid [15, 16, 32, 33]. The Western blotting results obtained in this study further proved that the cleavage of caspase-3 was promoted while the phosphorylation of AKT and ERK was inhibited, which indicates that the PI3K/AKT and MAPK/ERK pathways are also involved in the antitumour mechanism of chebulinic acid.

## Conclusion

Triphala is a phenolic-rich herbal formulation that is reputed for its anticancer activities. In this study, the obtained evidence indicates that chebulinic acid, as one of the major compounds in triphala, is required for the antitumour effects of triphala. The antineoplastic properties of chebulinic acid include anti-proliferation, pro-apoptosis, and anti-migration activities. The molecular mechanism is associated with the PI3K/AKT and MAPK/ERK pathways. These results suggest the therapeutic potential of chebulinic acid for human colorectal cancer. However, the detailed molecular mechanism of the antitumour effects of chebulinic acid remains to be clarified.

## Abbreviations

CCK: Cell counting kit
GATA: GATA-binding protein
HHDP: Hexahydrodiphenoyl
IC_50_: Half maximal inhibitory concentration
LSD: Least significant difference
MMP: Matrix metalloprotease
OD: Optical density
RGS: Regulator of G protein signalling
SD: Standard deviation
SMAD: Small mothers against decapentaplegic homologue
VEGF: Vascular endothelial growth factor
